# Crowding in real-time: an artificial intelligence-based model to manage crowding in the emergency department (CRAMMED Study)

**DOI:** 10.1186/s12873-026-01647-z

**Published:** 2026-06-17

**Authors:** E. E. Schippers, S. P. Willemsen, L. A. M. Collée, K. L. M. Koenraadt, S. Schol-Gelok

**Affiliations:** 1 Department of Emergency Medicine, Albert Schweitzer Ziekenhuis, Dordrecht, The Netherlands; 2 Department of Biostatistics, Erasmus Medical Centre, Rotterdam, The Netherlands; 3 Department of Quality, Safety and Innovation, Albert Schweitzer Ziekenhuis, Dordrecht, The Netherlands

**Keywords:** Emergency service, Hospital, Crowding, Ambulance diversion, Artificial Intelligence, Machine learning

## Abstract

**Background:**

Emergency department (ED) crowding is a global challenge with adverse effects on patient outcomes and staff well-being. Traditional crowding scores, such as the National ED Overcrowding Study (NEDOCS) score, have limited ability to predict imminent crowding. The aim of this study is to develop and validate a real-time, artificial intelligence (AI)-driven prediction model using a long short-term memory (LSTM) neural network to predict ED crowding, using ambulance diversion status as the reference standard and comparing performance with the (modified) NEDOCS score.

**Methods:**

In this single-centre, retrospective cohort study, we extracted parameters related to crowding from electronic health records and workforce data. Ambulance diversion status served as the reference standard for crowding. The LSTM model was trained and evaluated with walk-forward validation and performance was assessed using the area under the receiver operating characteristic curve (AUC). Model calibration was assessed using Brier score and expected calibration error (ECE).

**Results:**

The optimal LSTM model used the previous hour of data to predict one hour ahead, yielding an AUC of 0.84 (95% CI 0.81–0.86). Key features contributing to the predictive quality of our model included the number of pre-notified arrivals, patients in the yellow and orange triage categories, and the number of ED supervisors. Compared with the modified NEDOCS score (AUC of 0.73 (95% CI 0.70–0.76)), the LSTM model showed higher discriminative performance in internal validation within this single-centre study.

**Conclusions:**

Our AI-driven prediction model demonstrated good predictive performance for ED crowding in internal validation. These findings highlight the potential of real-time prediction models to support ED clinical workflow.

**Supplementary Information:**

The online version contains supplementary material available at 10.1186/s12873-026-01647-z.

## Background

Crowding occurs when the demand for emergency department (ED) or hospital services exceeds the available resources for adequate and timely patient care [1]. ED crowding is driven by various factors, including high patient inflow, prolonged throughput due to process constraints, and delayed outflow to inpatient wards [2–3]. Crowding is a global problem that has detrimental effects on both patients and healthcare workers. Delays in treatment, increased pain, discomfort, and higher mortality have been reported. For ED staff, crowding leads to an increase in work-related stress and burnout [4–6].

Various tools have been developed to predict and anticipate crowding in the ED [7–9]. The most commonly used crowding tool is the National ED Overcrowding Study (NEDOCS) scale. It consists of seven parameters that affect crowding and yields a continuous score ranging from 0 to 200, with standardised cut-off values defining levels of ED crowding. A score of > 100 indicates overcrowding [8, 9]. Multiple studies show that the NEDOCS scale demonstrates good discriminatory power for ED crowding [5, 8–11]. However, the NEDOCS scale turns out to be inadequate in extremely high-volume EDs and does not always correspond with ED staff’s perception of crowding [12, 13]. Furthermore, it has limited ability to predict near-future crowding and recommended threshold values may not be generalisable [14, 15]. To overcome these disadvantages, the modified NEDOCS (mNEDOCS) was created. The mNEDOCS uses the number of patients with the highest triage level instead of patients on mechanical ventilation. There was a strong correlation between perceived crowding by ED staff in the Netherlands and the mNEDOCS [16]. The parameters used in the (m)NEDOCS are predominantly patient flow and patient occupancy measures, which do not necessarily correspond with the actual workload for ED staff. It ignores multiple patient and staff related factors, even though strong correlations were found between crowding and staff-related factors such as the on-duty nurse count [12].

Ambulance diversions are commonly used as a proxy for ED crowding, as the need to divert patients indicates high patient load. They also serve to reduce crowding by directing incoming patients to hospitals with more available capacity. In the Netherlands, ambulance diversions are registered and coordinated through the National Acute Coordination Platform, a real-time nationwide application that monitors ED capacity using traffic light colours. The system also records the underlying reason for the ambulance diversion, such as high patient inflow or delayed outflow. An ambulance diversion is initiated by the attending ED physician or nurse in charge when all reasonable measures to improve ED throughput have proven insufficient. Each colour corresponds to the current ED capacity: green indicates normal ED capacity, orange signals impending ED crowding, red reflects active ambulance diversion and black denotes full closure due to a calamity, such as a major technical problem [17].

It is essential to find evidence-based measures of ED crowding to quantify, predict, and prevent it. Previous studies have explored prediction models for ED occupancy [18], but their use in predicting perceived ED crowding remains limited. This study aimed to develop a real-time, artificial intelligence (AI)-driven model to predict ED crowding, using current or impending ambulance diversions status as a proxy.

## Methods

### Study design, setting and study population

This single-centre, retrospective cohort study was executed in the Albert Schweitzer hospital, Dordrecht, The Netherlands: a non-academic, high-volume teaching hospital. The hospital receives an average of 31,000 ED patients annually providing a sufficiently large cohort for model development. All patients presenting to the ED in 2023 were eligible, except those who had explicitly opted out of the research use of their medical data. This study was reported in accordance with the STROBE Statement for observational studies (Appendix A). The hospital’s institutional review board approved the study and granted a waiver of informed consent.

### Outcomes and measurements

The primary aim of this study was to develop and internally validate a real-time, AI-driven long short-term memory (LSTM) model [19]. Secondary aims were to compare the model’s performance with that of the mNEDOCS and to quantify the contribution of individual parameters to the model’s predictive performance. ED crowding was defined as a binary outcome based on ambulance diversion status. Time points were labelled as positive (1) if the ED was in, or entered, an ambulance diversion state (orange or red) within the specified prediction horizon, and negative (0) otherwise (green). Data were aggregated at five-minute intervals, and each time point was labelled by evaluating whether ambulance diversion occurred within the subsequent prediction window. For each prediction horizon (e.g. 1, 2, and 3 h), the model estimated the probability that the ED would be in a diversion state at any time within that future time window. Table 1 presents an overview of the mNEDOCS.

**Table 1:** Parameters included in the modified national emergency department crowding score (mNEDOCS)

Total number of patients in the ED
Total number of ED beds
Total number of admits in the ED
Total number of hospital beds
Longest admit boarding time in the ED
Hours of longest wait in the waiting room
Number of patients being resuscitated or assigned the highest acuity level

Overview of parameters included in the modified NEDOCS score

### Identification of ED crowding parameters

After consulting a health sciences librarian, we conducted a structured literature search in PubMed and Embase to identify studies on ED crowding parameters. The search covered the period from January 2000 until February 2024 (time of search) and yielded 2538 results. Titles and abstracts were screened for relevance before full-text review. Twelve articles focusing on ED crowding determinants were selected, from which we compiled a preliminary list of 36 individual parameters (full search strategies in Appendix B). We then consulted a panel of two emergency physicians and three nurses from our emergency department to evaluate the contribution of each parameter to crowding in our ED.

The panel considered both their professional judgment as well as their daily perception of ED crowding to ensure the selection reflected the practical aspects of ED crowding. Parameters were selected through expert consensus following group discussion. To ensure feasibility of real-time application and avoid duplication, parameters that were redundant, not measurable, or not routinely available within the hospital information systems were excluded. This process resulted in a refined list of seventeen parameters. Table 2 provides a detailed description of the selected parameters.

**Table 2:** Definitions of selected emergency department parameters

Parameter	Unit	Definition
ED occupancy rate	%	(Number of occupied ED beds/total ED capacity) x 100%
ED volume	n	Number of patients physically present in the ED (treatment bays, waiting rooms, hallway)
Total ED length of stay	min	Time from ED registration to ED departure
Number of pre-notified arrivals	n	Number of pre-notified ED patients (expected but not yet present)
Longest admit boarding time	min	Maximum time from admission order to inpatient ward transfer
Number of boarding patients in ED	n	Number of patients in the ED awaiting admission (“boarding”)
Total occupied hospital beds	n	Number of occupied inpatient hospital beds
Number of ED patients in isolation	n	Number of patients in the ED under isolation precautions
Time to radiology	min	Time from order placement to imaging availability
Elapsed time to triage	min	Delay beyond permitted triage interval (actual triage time minus 15 min)
Patients in each triage category	n	Number of patients per Manchester Triage System category (Red, Orange, Green, Yellow or Blue)
Elapsed time to treatment / physician assessment	min	Delay beyond target assessment time (depending on triage category)^a^
Elderly high-risk screening (≥ 70 years)	n	Number of patients aged ≥ 70 scoring “high risk of adverse outcomes” on APOP screener
Patients aged 0–3 years	n	Number of pediatric patients in aged 0–3 years
Staff availability: ED nurses	n	Number of nurses on duty
Staff availability: ED medical doctors	n	Number of junior doctors or specialty trainees on duty
Staff availability: ED supervisors	n	Number of ED physicians on duty

^a^Permitted time to assessment according to Manchester Triage System categories (Red: 0 min; Orange: < 10 min; Yellow: < 30 min; Green: < 90 min; Blue: < 120 min)

### Data collection

Data of the selected seventeen parameters were retrieved to develop the predictive model. Patient-related data were extracted from the electronic patient record (EPR). The ED staff’s occupation rate was extracted from ORTEC workforce scheduling software. The mNEDOCS along with the colours indicating ambulance diversion were extracted from the National Acute Care Coordination Platform. All data were extracted at five-minute intervals. Missing values were handled using forward-filling, carrying the last observed value forward. All variables included in the model were derived from routinely recorded timestamps and represent patient-level time intervals as defined in Table 2. These variables were subsequently aggregated at each time point to generate system-level model inputs. All patients were included to avoid selection bias. Time-based variables were calculated only when the relevant timestamps were present in routine clinical care for each patient-timepoint.

The data were analysed using Statistical Package for the Social Sciences (SPSS^®^) software version 25 or higher, R software version 4.3.3 or higher, TensorFlow (Google LLC) version 2.17.0, and the Keras Application Programming Interface (version 3.6.0), running on Python (version 3.11.8).

### Model development and performance evaluation

We developed an LSTM model to analyse time-series data and predict the probability of ED crowding at predefined future time points. The model used sliding windows of 2–5 hourly time steps, each representing the ED state at that time. Variables originally recorded at higher temporal resolution were downsampled to hourly intervals. For time-stamped patient-level variables, both the current status and elapsed time since the last status change were calculated at each time point. To improve performance and reduce overfitting, input normalization and regularization techniques were applied. Hyperparameters were tuned using random search over 36 configurations, varying learning rate, LSTM architecture, and L1 and L2 regularisation terms. Models were trained using the Adam optimiser to minimise binary cross-entropy (logistic) loss, with a batch size of 32 and a maximum of 60 epochs. For each combination of hyperparameters, a separate LSTM model was constructed, trained, and evaluated using walk-forward validation. Data were ordered chronologically, with the initial training set comprising 60% of the available sequences. The remaining data were evaluated in four sequential validation folds. In each fold, the training window was expanded forward in time, and the model was retrained and tested on the subsequent unseen period. This procedure was repeated until all folds were evaluated on unseen future data. The final model was selected based on the hyperparameter configuration with the lowest mean logistic loss across folds. Appendix C provides a detailed description of the final model architecture. Parameters with a skewed distribution are reported as median with interquartile range (IQR), whereas normally distributed parameters are reported as mean with standard deviation (SD).

Model performance was evaluated using the area under the receiver operating characteristic curve (AUC) for each prediction horizon, which served as the primary metric for selection of the final model. As expected, AUC varied across prediction horizons depending on the number of hours ahead being predicted. Formal statistical comparisons of AUC between the LSTM model and the mNEDOCS score were not performed, as both models were evaluated on the same time-series dataset and are therefore not statistically independent.

Model calibration was assessed using the Brier score, expected calibration error (ECE), and calibration slope. The Brier score reflects the mean squared difference between predicted probabilities and observed outcomes, while the ECE quantifies the average absolute difference between predicted and observed event rates across probability bins. The calibration slope was estimated using logistic recalibration. To evaluate clinical applicability at decision thresholds, we determined the probability threshold corresponding to an 85% sensitivity. At this threshold, specificity, positive predictive value, negative predictive value, and overall error rate were calculated.

Parameter importance was estimated using a permutation-based approach. Each input feature was randomly shuffled across the validation set while keeping all other features unchanged. The resulting change in model performance (ΔAUC), was recorded as the importance score. Positive scores indicated that the feature contributed meaningful information, whereas negative scores suggested potential noise or overfitting.

## Results

### Demographic and clinical characteristics

During the predefined study period (January 2023 until December 2023) a total of 30,146 ED visits were registered. Data from 27,864 ED visits were included after excluding the data of patients who objected to the use of their medical data. The study population included 1729 children aged 0–3 years (6.21%) and 11,224 patients aged ≥ 70 years (40.3%). A total of 5252 elderly patients (46.8%) were screened using the APOP screener, of whom 2538 (48.3%) were identified as high risk.

Triaged patients were predominantly in the yellow (46.5%) and orange (29.6%) categories. The median length of stay (LOS) in the ED was 206 min (P25-P75: 144–277) and median daily ED volume was 75 patients (P25-P75: 70–83). In total 765 h of impending ambulance diversions and 310 h of complete ambulance diversions were recorded, corresponding to 12.3% of the time. The median duration of complete ambulance diversions was 59 min (P25-P75: 39–91). The majority (46.1%) of ambulance diversions were initiated due to peaks in patient arrivals, followed by limited ED outflow (34.6%). The median mNEDOCS was 44.0 (P25-P75:2-170). Most parameters had minimal missing data (< 2%). The mNEDOCS had 5.87% missing values due to occasional connectivity issues with the application, causing temporary gaps in the measurements. Three potentially relevant parameters could not be included in the model due to data limitations. ED occupancy could not be reliably derived because it was not possible to distinguish between patients occupying a bed and those in the ED waiting room or elsewhere. Boarding time and the total number of boarding patients were excluded due to unreliable documentation of the exact times when admission and discharge decisions were made in the ED. Detailed demographic and ED characteristics are presented in Table 3.

**Table 3:** Baseline demographic and clinical characteristics

Characteristic	Total ED visits (*N* = 27,864)
Demographics and clinical characteristics	
Age, median (IQR), years	61 (34–76)
0–3 years, n (%)	1729 (6.21%)
≥ 70 years, n (%)	11,224 (40.3%)
Elderly screened with APOP, n (% of elderly)	5252 (46.8%)
High-risk APOP among screened elderly, n (%)	2538 (48.3%)
Isolation precautions, n (%)	3461 (12.4%)
Patients in red triage category, n (%)	574 (2.06%)
Patients in orange triage category, n (%)	8250 (29.6%)
Patients in yellow triage category, n (%)	12,966 (46.5%)
Patients in green triage category, n (%)	5767 (20.7%)
Patients in blue triage category, n (%)	137 (0.49%)
Missing triage category, n (%)	170 (0.61%)
Emergency department crowding measures	
Number of pre-notified arrivals, median (IQR)	3 (2–5)
Daily ED volume, median (IQR)	75 (70–83)
Hourly ED volume, median (IQR)	9 (4–15)
Length of stay, median (IQR), min	206 (144–277)
Number of occupied hospital beds, mean (SD)	268 (24.9)
Time to triage, median (IQR), min	13 (7–21)
Time to radiology, median (IQR), min	90 (49–178)
Daily number of supervisors on duty, mean (SD)	1.77 (0.23)
Daily number of medical doctors on duty, median (IQR)	3.5 (3.33–3.83)
Daily number of nurses on duty, median (IQR)	7.63 (7.25–8.04)
Impending ambulance diversions, hours (% of total time)	765 (8.73%)
Ambulance diversions, hours (% of total time)	310 (3.53%)
mNEDOCS, median (IQR)	44.0 (2-170)

Baseline demographic and clinical characteristics of ED visits and ED crowding measures (January-December 2023). ED occupancy, time to treatment, total boarding patients and boarding time were inconsistently recorded in the electronic patient record and therefore excluded from descriptive analyses due to unreliable data.

## Model selection, performance, calibration and feature importance

**Fig. 1:**
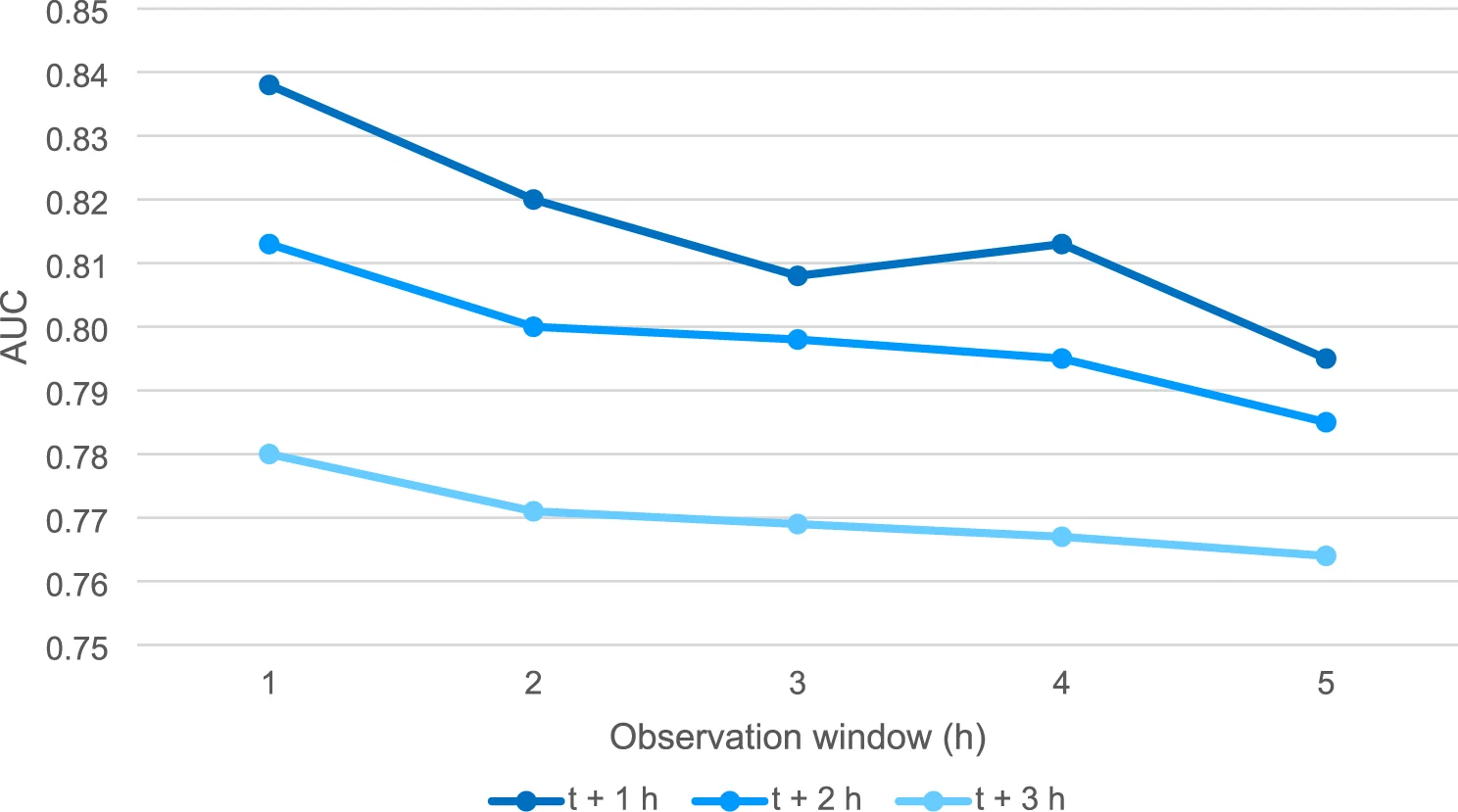
Model performance of the long short-term memory (LSTM) model. Figure 1 Model performance, expressed as area under the receiver operating characteristic curve (AUC), across different observation windows and prediction horizons. The observation window (h) represents the number of preceding hours of data used as model input. The prediction horizons (t + 1 h, t + 2 h, t + 3 h) represent the model’s prediction for the upcoming 1–3 h.

The best performing model used a learning rate of 0.0003, a single LSTM layer with eight hidden units, and a dense sigmoid output layer. An L2 regularisation penalty of 0.0001 was applied, while no L1 regularisation was used. No regularisation was applied to the recurrent connections, which yielded the best performance. Model performance was evaluated using the AUC. Figure 1 shows AUCs for different numbers of past observations and prediction horizons ranging from one to three hours ahead. The highest AUC was 0.84 (95% CI 0.81–0.86) when using one past observation to predict ED crowding at a one-hour prediction horizon.

On the same dataset, the mNEDOCS achieved an AUC of 0.73 (95% CI 0.70–0.76) for the same observation window and prediction horizon. Model calibration showed a Brier score of 0.09, ECE 0.046, and a calibration slope of 1.1. At the probability threshold corresponding to ≥ 85% sensitivity (5.32%), specificity was 46%, positive predictive value 17%, negative predictive value 96%, and overall error rate 49%.

**Fig. 2:**
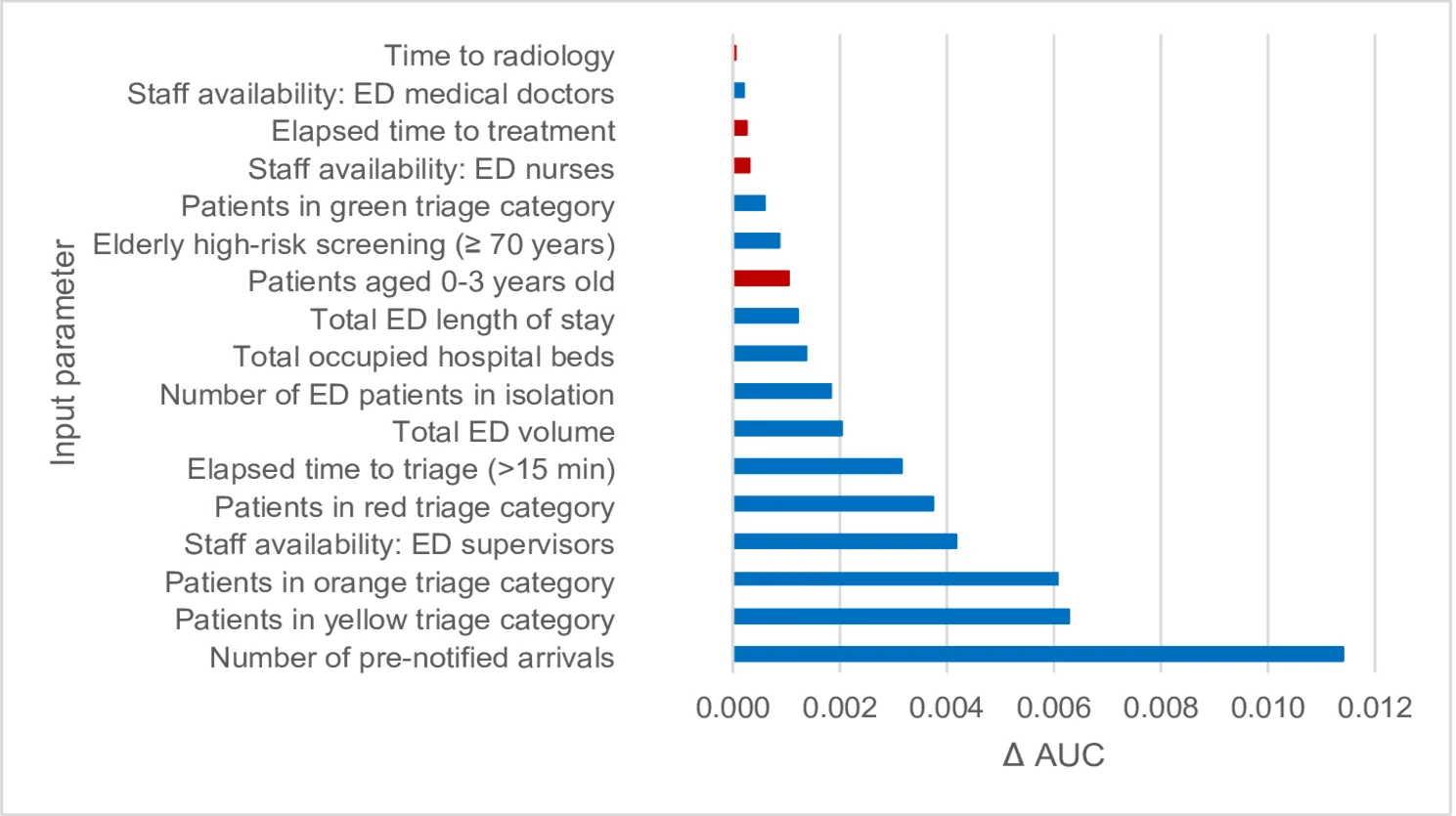
Feature importance for the long short-term memory (LSTM) model. Figure 2. Importance of input features for the long short-term memory (LSTM) model, expressed as the change in the area under the receiver operating characteristic curve (ΔAUC). Blue bars indicate positive contributions to model performance, red bars indicate negative contributions or noise.

Finally, feature importance was assessed to determine the individual contribution of each parameter to the predictive performance of the LSTM model (Fig. 2). The number of pre-notified arrivals, number of patients in yellow and orange triage categories and number of ED supervisors contributed most. In contrast, time to radiology, elapsed time to treatment, number of ED nurses and patients aged 0–3 years showed negative contributions.

## Discussion

In this study, we developed a novel real-time AI-driven prediction model that showed higher discriminative performance than the mNEDOCS in internal validation. To our knowledge, few studies have directly compared an LSTM neural network for ED crowding with traditional crowding measures using real-world hospital and ambulance diversion data. Traditionally, static crowding scores such as the NEDOCS are widely used, whereas more complex machine learning algorithms are rarely implemented in routine ED operations [20]. Several reviews have shown the potential of AI to optimize ED flow, for example by improving waiting time, risk assessment, and predicting hospital admissions [20–23]. Nas et al. used real ED arrival data in a simulation model to minimize LOS, showing a 7% reduction in LOS [24].

Similarly, Vural et al. developed a time-series model to predict ED waiting room occupancy, achieving a mean absolute error of 4.19 patients [25]. In addition, Song et al. developed machine learning models to predict LOS and decisions regarding patient disposition (e.g. admission or discharge), achieving strong discriminative performance with AUC values of 0.862–0.878 for LOS and 0.839–0.863 for disposition predictions [26]. These studies highlight the potential of machine learning for operational decision-making in the ED. Building on this work, our study extends machine learning approaches to predict imminent ED crowding.

While our findings suggest that the LSTM model can more accurately reflect imminent ED crowding than the mNEDOCS in our hospital setting, this comparison should be interpreted cautiously. The mNEDOCS is a static score, whereas the LSTM neural network is trained on site-specific, high-frequency, real-time ED data, capturing temporal patterns and complex nonlinear relationships in the data that the mNEDOCS cannot. Therefore, the model’s performance is strongly context-dependent and implementation in other settings would require local retraining to account for differences in workflow, staffing, and operational dynamics. Furthermore, some features in the LSTM model correspond to parameters included in the mNEDOCS, meaning the models are not fully independent. At the same time, certain features were deliberately defined differently to better capture ED dynamics. For instance, pre-notified arrivals were used instead of the mNEDOCS parameter longest wait in the waiting room. Unlike waiting room hours, which reflect ED crowding that has already occurred, pre-notified arrivals provide a forward-looking signal of incoming patients, enabling the model to anticipate ED crowding more effectively.

When evaluating different prediction horizons, the highest discriminative performance was observed for the 1-hour observation window predicting 1 h ahead. As expected, predictive performance gradually decreased with longer prediction horizons. Although shorter horizons yielded the best performance, a modest reduction in AUC may be acceptable if a longer lead time improves operational decision-making. The threshold analysis further highlights the inherent trade-off between sensitivity and specificity. Prioritising sensitivity improves detection of (impending) ED crowding but increases false positives, underscoring the need to select operating thresholds and prediction horizons based on clinical utility. However, as this study was designed for model development and internal validation purposes, no conclusions can yet be drawn regarding optimal thresholds or direct use for operational decision-making in clinical practice.

Permutation-based feature importance analysis revealed clear differences in the contribution of parameters to the model’s predictive performance. Input-related features, particularly pre-notified arrivals and ED volume, contributed most to LSTM performance, emphasizing real-time inflow in anticipating ED crowding. In general, LSTM models benefit from features with clear temporal patterns and high-frequency measurements, as these allow the network to learn sequential trends effectively.

In our study, features with these characteristics included the number of pre-notified arrivals, total ED volume, and patients in the yellow and orange triage categories. Among these, patients in the yellow and orange categories contributed more to predictive accuracy than red-triaged patients, reflecting that these groups are larger and more temporally variable, and thus more informative for capturing dynamic ED crowding patterns. Similarly, the number of supervising physicians likely reflects anticipated workload patterns within the local staffing and scheduling system. Higher staffing levels are scheduled during historically busier periods rather than indicating a direct causal effect on ED crowding.

Various other process-related parameters, such as time to triage and time to treatment, depend on manual registration and may partly reflect documentation practices and workload. Their observed importance suggests they serve as indicators of system pressure rather than direct drivers of ED crowding. Overall, these findings indicate that the parameters should be interpreted as system-state indicators of ED crowding dynamics, rather than evidence of a causal relationship.

Several limitations of this study should be acknowledged. First, the model is inherently site-specific, as it was developed and internally validated in a single-centre dataset reflecting local operational workflows and definitions of ED crowding. In addition, we relied partly on expert opinion for the final selection of parameters, potentially introducing selection bias.

Second, although the model incorporated multiple inflow, throughput and output parameters of ED crowding, other key factors such as temporal and climatic parameters were not included, possibly limiting the model’s responsiveness to sudden surges in ED demand.

Furthermore, although available hospital capacity was included as a model feature, outflow-related measures such as boarding time and total number of boarding patients could not be reliably incorporated due to inconsistent documentation. Consequently, the model’s predictive performance may have been influenced by the absence of these features. Forward-filling was used to handle missing values, which may have introduced residual bias. Such smoothing of temporal fluctuations could potentially have led to misestimation of short-term dynamics, thereby affecting model performance.

Finally, the traffic light system for ambulance diversions was used as the reference standard. Although we believe it is the best available reference measure in our setting, as it reflects real-time operational decision-making during periods of system strain, it may not capture all dimensions of ED crowding. As stated by Baan-Kooman et al. [16], the decision to announce an ambulance diversion is made by individuals, thereby reflecting perceived ED crowding, a subjective measure. Consequently, our model may in part capture staff decision-making patterns instead of true ED crowding. In addition, ambulance diversion is not uniformly implemented across healthcare systems. It is more commonly used in urban or hospital-dense regions, whereas in rural settings, formal diversion policies are often not applied. Therefore, the generalisability of our findings may be limited to settings where ambulance diversions are routinely used as an indicator of ED crowding.

## Conclusions

In conclusion, our LSTM model demonstrated good predictive performance for ED crowding and showed higher discriminative performance than the mNEDOCS score in internal validation within our hospital. It could provide early warnings of impending ED crowding, enabling short-term responses such as prioritisation of patient flow and redistribution of staff within a shift.

Given that the model was trained on highly site-specific operational data, future studies should focus on evaluating how locally retrained and validated versions of the model perform in different ED settings, determining optimal prediction thresholds, and assessing how such models can be integrated into real-time clinical practice. We strongly recommend efforts to improve user-friendliness to facilitate integration into daily ED workflow and promote clinical adoption by users [22].

## Supplementary Information

Below is the link to the electronic supplementary material.

Supplementary Material 1

Supplementary Material 2

Supplementary Material 3

## Data Availability

The datasets generated and/or analysed during the current study are not publicly available due to data protection regulations and institutional data governance policies. The datasets are available from the corresponding author on reasonable request.

## Abbreviations

AI: Artificial Intelligence
APOP: Acute Presenting Older Patient (screener)
AUC: Area Under the Curve
CI: Confidence Interval
CRAMMED: Crowding in Real–time: an Artificial intelligence–based Model to Manage crowding in the Emergency Department
ED: Emergency Department
EPR: Electronic Patient Record
IQR: Interquartile Range
LOS: Length of Stay
LSTM: Long Short–Term Memory
mNEDOCS: Modified National Emergency Department OverCrowding Score
MTS: Manchester Triage System
NEDOCS: National Emergency Department Overcrowding Study
P25: P75–25th–75th percentile
ROC: Receiver Operating Characteristic
SD: Standard Deviation
SPSS: Statistical Package for the Social Sciences
STROBE: Strengthening the Reporting of Observational Studies in Epidemiology
