# Supplementary material for: Crowding in real-time: an artificial intelligence-based model to manage crowding in the emergency department (CRAMMED Study)
Source: BMC Emerg Med. 2026 Jun 17;26:232. doi: 10.1186/s12873-026-01647-z (PMC13520244; doi:10.1186/s12873-026-01647-z)
Supplement: Supplementary file 1 [file 12873_2026_1647_MOESM1_ESM.docx]

**Appendix B. Literature search strategy**

A structured search was conducted to identify articles discussing parameters contributing to emergency department (ED) crowding. The following search strategy was applied in PubMed and Embase:

| **Search terms** | "emergency service, hospital"[MeSH Terms] OR "hospital emergency service*"[Title/Abstract] OR "emergency hospital service*"[Title/Abstract] OR "hospital emergency room*"[Title/Abstract] OR  "accident and emergency department*"[Title/Abstract] OR "emergency ward*"[Title/Abstract] OR "emergency department*"[Title/Abstract] OR "emergency unit*"[Title/Abstract] OR "emergency services utilization*"[Title/Abstract] OR "emergency outpatient unit*"[Title/Abstract] AND "Crowding"[Mesh] OR "crowding"[Title/Abstract] OR "crowd*"[Title/Abstract] OR "overcrowd*"[Title/Abstract] OR "overcrowding*"[Title/Abstract] |
| --- | --- |
|  |  |
| **Keywords** | ‘emergency service, hospital’, ‘hospital emergency service’, ‘emergency hospital service’, ‘hospital emergency room’, ‘accident and emergency department’, ‘emergency ward’, ‘emergency department’, ‘emergency unit’, ‘emergency room’, ‘emergency services utilization’, ‘emergency outpatient unit’ AND ‘crowding’, ‘crowd’, ‘overcrowd’, ‘overcrowding’. |
| **Filters** | ‘Full text’, ‘Systematic Review’, ‘Review’, ‘Meta-Analysis’. |
| **Period** | January 2000 – February 2024. |
| **Results** | 2,538 articles. |
| **Inclusion criteria** | Articles addressing parameters that contribute to ED crowding. |
| **Exclusion criteria** | Articles not available in full text, not in English, or focusing solely on consequences of overcrowding or interventions rather than contributing factors. |
