# Supplementary material for: Crowding in real-time: an artificial intelligence-based model to manage crowding in the emergency department (CRAMMED Study)
Source: BMC Emerg Med. 2026 Jun 17;26:232. doi: 10.1186/s12873-026-01647-z (PMC13520244; doi:10.1186/s12873-026-01647-z)
Supplement: Supplementary file 2 [file 12873_2026_1647_MOESM2_ESM.docx]

**Appendix C. LSTM model architecture and training details**

| **Section** | **Component** | **Description** |
| --- | --- | --- |
| **Overview** | - | Recurrent neural network using long short-term memory (LSTM) layers to capture temporal patterns in longitudinal patient data. Implemented in TensorFlow (Google LLC) and Keras deep learning framework. TensorFlow handles efficient numerical optimization on modern hardware and Keras provides a user-friendly and reproducible framework for defining, training, and validating neural network models. |
| **Inputs and Outputs** | Input | Multivariate time-series data. Each observation consists of a fixed-length sliding window of 2–5 hourly time steps derived from downsampled five-minute interval data. Shape: (number of time steps × number of features). |
|  | Output | The model outputs predicted probabilities of ED crowding at predefined prediction horizons (t+1, t+2, ..., t+*H*). Each output node corresponds to a horizon and produces a value between 0 and 1. |
| **Model Architecture** | Input Normalization | All time-varying input features were standardized using a normalization layer fitted on training data only, ensuring numerical stability and preventing large-scale features from dominating model optimization. |
|  | Stacked LSTM Layers | The core is a stack of one or more LSTM layers with 2–8 hidden units each. Intermediate layers return full sequences to allow deeper temporal abstraction; the final layer returns a single summary representation of the input window. LSTM activation function: hyperbolic tangent (*tanh*). L1 and L2 penalties applied to LSTM weights to reduce overfitting. |
|  | Output Layer | Fully connected (dense) layer with *H* units (prediction horizon). Sigmoid activation for probability output to create an outcome between 0-1 for each future time point. L2 penalty applied to the output layer to reduce overfitting. |
| **Training Details** | Loss Function | Binary cross-entropy (logistic) loss computed across all prediction horizons. |
|  | Optimizer | Adam optimizer (default setting). |
|  | Training Settings | Training was performed using a batch size of 32 and a maximum of 60 epochs. |
|  | Validation | Walk-forward (temporal) validation scheme: train on early portion of time-ordered data, expand training window, evaluate on future unseen observations. Repeated over multiple folds to obtain stable performance estimates. To prevent overfitting and unnecessary training, early stopping was employed based on validation loss, with restoration of best-performing model weights. |
|  | Evaluation Metrics | Model performance: area under the receiver operating characteristic curve (AUC). Model calibration: Brier score, expected calibration error, and calibration slope. Threshold-based performance metrics: sensitivity, specificity, positive predictive value, and negative predictive value. |
|  | Hyperparameters | Random search over 36 configurations (learning rate, number of LSTM layers, number of hidden units (2–8), L1/L2 penalty sizes). |
