# Supplementary material for: Crowding in real-time: an artificial intelligence-based model to manage crowding in the emergency department (CRAMMED Study)
Source: BMC Emerg Med. 2026 Jun 17;26:232. doi: 10.1186/s12873-026-01647-z (PMC13520244; doi:10.1186/s12873-026-01647-z)
Supplement: Supplementary file 3 [file 12873_2026_1647_MOESM3_ESM.docx]

**Appendix A. STROBE (Strengthening the Reporting of Observational Studies in Epidemiology) Statement for observational studies**

|  | | Item No. | Recommendation | Page  No. | | | Relevant text from manuscript |
| --- | --- | --- | --- | --- | --- | --- | --- |
| **Title and abstract** | | 1 | (*a*) Indicate the study’s design with a commonly used term in the title or the abstract | 1 | | | Abstract: “single-centre, retrospective cohort study” |
|  |  |  | (*b*) Provide in the abstract an informative and balanced summary of what was done and what was found | 1 | | | Abstract: Background, objectives, methods, results and conclusions |
| Introduction | | | | | | |  |
| Background/rationale | | 2 | Explain the scientific background and rationale for the investigation being reported | 2, 3 | | | Introduction: first paragraphs explain crowding, current situation, and need for a predictive model. |
| Objectives | | 3 | State specific objectives, including any prespecified hypotheses | 3 | | | Introduction, last sentence: “This study aimed to develop a real-time, artificial intelligence…” |
| Methods | | | | | | |  |
| Study design | | 4 | Present key elements of study design early in the paper | 3 | | | Methods, first sentence: “This single-centre, retrospective cohort study was executed…” |
| Setting | | 5 | Describe the setting, locations, and relevant dates, including periods of recruitment, exposure, follow-up, and data collection | 3 | | | Methods: Study design, setting and study population. |
| Participants | | 6 | (*a*) Give the eligibility criteria, and the sources and methods of selection of participants. Describe methods of follow-up | 3 | | | Methods: all ED patients in 2023 included, except those who explicitly opted out of data use. |
|  |  |  | (*b*) For matched studies, give matching criteria and number of exposed and unexposed |  | | | N/A |
| Variables | | 7 | Clearly define all outcomes, exposures, predictors, potential confounders, and effect modifiers. Give diagnostic criteria, if applicable | 4-6 | | | Methods: Outcomes and measurement; Identification of ED crowding parameters; Table 1 |
| Data sources/ measurement | | 8 | For each variable of interest, give sources of data and details of methods of assessment (measurement). Describe comparability of assessment methods if there is more than one group | 6 | | | Methods: Data collection |
| Bias | | 9 | Describe any efforts to address potential sources of bias | 4, 6 | | | Methods: “To minimize selection bias…” and “Missing values were handled using forward-filling…” |
| Study size | | 10 | Explain how the study size was arrived at | 3 | | | Methods: “The hospital receives an average of 31,000 ED patients annually…” |
| Quantitative variables | | 11 | Explain how quantitative variables were handled in the analyses. If applicable, describe which groupings were chosen and why | 6, 7 | | | Methods: “Data were aggregated at five-minute intervals, and each time point was labelled....” |
| Statistical methods | | 12 | (*a*) Describe all statistical methods, including those used to control for confounding | 6, 7 | | | Methods: “All data were used in aggregated form and extracted at five-minute intervals.” “Input normalization and regularization techniques were applied.” |
|  |  |  | (*b*) Describe any methods used to examine subgroups and interactions | 7 | | | Methods: Model development and performance evaluation |
|  |  |  | (*c*) Explain how missing data were addressed | 6 | | | Methods: “Missing values were handled using forward-filling…” |
|  |  |  | (*d*) If applicable, explain how loss to follow-up was addressed |  | | | N/A |
|  |  |  | (*e*) Describe any sensitivity analyses |  | | | N/A. Sensitivity analyses were not performed, as all available ED patients were included and missing data were minimal and handled via forward-filling. |
| Results | | | | | | | |
| Participants | 13 | (a) Report numbers of individuals at each stage of study—eg numbers potentially eligible, examined for eligibility, confirmed eligible, included in the study, completing follow-up, and analysed | | | 8 | | Results: “…30,146 ED visits were registered. Data from 27,864 ED visits were included…” |
|  |  | (b) Give reasons for non-participation at each stage | | |  | | N/A |
|  |  | (c) Consider use of a flow diagram | | |  | | N/A |
| Descriptive data | 14 | (a) Give characteristics of study participants (eg demographic, clinical, social) and information on exposures and potential confounders | | | 8-10 | | Results: Demographic and clinical characteristics; Table 2. |
|  |  | (b) Indicate number of participants with missing data for each variable of interest | | | 8-10 | | Results: Demographic and clinical characteristics; Table 2. |
|  |  | (c) Summarise follow-up time (eg, average and total amount) | | |  | | N/A |
| Outcome data | 15 | Report numbers of outcome events or summary measures over time | | | 9-12 | | Results: Table 3; Figure 1; Figure 2 |
| Main results | 16 | (*a*) Give unadjusted estimates and, if applicable, confounder-adjusted estimates and their precision (eg, 95% confidence interval). Make clear which confounders were adjusted for and why they were included | | | 10, 11 | | Results: AUC value with 95% confidence intervals reported. |
|  |  | (*b*) Report category boundaries when continuous variables were categorized | | | 5, 6 | | Continuous variables were analysed as continuous; categorization was only applied for predefined age groups (0–3 years, ≥70 years) and triage categories |
|  |  | (*c*) If relevant, consider translating estimates of relative risk into absolute risk for a meaningful time period | | |  | | N/A |
| Other analyses | 17 | Report other analyses done—eg analyses of subgroups and interactions, and sensitivity analyses | | |  | | N/A |
| Discussion | | | | | | | |
| Key results | 18 | Summarise key results with reference to study objectives | | | 12 | Discussion: first paragraph; summary of main findings. | |
| Limitations | 19 | Discuss limitations of the study, taking into account sources of potential bias or imprecision. Discuss both direction and magnitude of any potential bias | | | 14 | Discussion: limitations paragraph: single-centre design, expert selection bias, missing data, subjective outcome. | |
| Interpretation | 20 | Give a cautious overall interpretation of results considering objectives, limitations, multiplicity of analyses, results from similar studies, and other relevant evidence | | | 12-15 | Discussion: comparative paragraph referencing multiple studies | |
| Generalisability | 21 | Discuss the generalisability (external validity) of the study results | | | 12, 13, 16 | Discussion: limitations: generalizability limited to single-site data. | |
| Other information | |  | | | | | |
| Funding | 22 | Give the source of funding and the role of the funders for the present study and, if applicable, for the original study on which the present article is based | | | 18 | Funding: funding for protected research time for the first author. | |
